# Curcumin: reclaiming the lost ground against cancer resistance

**DOI:** 10.20517/cdr.2020.92

**Published:** 2021-06-19

**Authors:** Siraj Shaikh, Javed Shaikh, Yusufi Sadia Naba, Kailas Doke, Khursheed Ahmed, Mujahid Yusufi

**Affiliations:** ^1^Post-Graduate Department of Chemistry and Research Center, Abeda Inamdar Senior College of Arts, Science and Commerce (Affiliated to SPPU), Pune 411001, India.; ^2^Advanced Scientific Research Laboratory, Azam Campus, Pune 411001, India.

**Keywords:** Curcumin, drug-resistant cancer, signaling pathways, clinical trials

## Abstract

Curcumin, a polyphenol, has a wide range of biological properties such as anticancer, antibacterial, antitubercular, cardioprotective and neuroprotective. Moreover, the anti-proliferative activities of Curcumin have been widely studied against several types of cancers due to its ability to target multiple pathways in cancer. Although Curcumin exhibited potent anticancer activity, its clinical use is limited due to its poor water solubility and faster metabolism. Hence, there is an immense interest among researchers to develop potent, water-soluble, and metabolically stable Curcumin analogs for cancer treatment. While drug resistance remains a major problem in cancer therapy that renders current chemotherapy ineffective, curcumin has shown promise to overcome the resistance and re-sensitize cancer to chemotherapeutic drugs in many studies. In the present review, we are summarizing the role of curcumin in controlling the proliferation of drug-resistant cancers and development of curcumin-based therapeutic applications from cell culture studies up to clinical trials.

## Introduction

Challenges posed by cancer have always been a notch higher than the advancements in chemotherapy. Treatment of cancer remains one of the most important concerns worldwide including United States. As per the estimates of American Chemical Society, United States may witness 1,806,590 new cancer cases and 606,520 deaths caused by cancer in 2020^[1]^. Currently employed chemotherapeutic regimens offer highly desired respite against cancer; nevertheless the resistance to anticancer drugs is the problem, which needs to be addressed on priority. Research in the area of drug resistance has revealed the complexity of the process that may involve alteration of drug targets, genetic differences and tumor heterogeneity. Drug resistance can be induced through numerous mechanisms such as increased drug efflux, suppression of apoptosis, altered drug metabolism, amplification of oncogene and epigenetic factors^[2]^. A drug-resistant cancer is labelled as Multi-Drug Resistant (MDR) cancer when it demonstrates resistance to two or more anticancer drugs. Identification of multi-drug resistance warrants better therapeutic strategies for successful cancer treatment.

In recent times, natural products have received immense attention due to their low toxicity, ability to negotiate with multiple targets implicated in cancer as well as their efficacy against elimination of cancer stem cells. Natural products-inspired novel lead compound account for almost 50% over the past few decades^[3]^. The naturally occurring compounds, especially phytochemicals and their synthetic conjugates^[4-6]^ have been widely investigated to explore the therapeutic potential for the prevention and treatment of cancer, whereas recent studies have also established their role in chemo-sensitizing cancer cells to overcome MDR^[7-12]^.

Curcumin is one such phytochemical which has been extensively studied. It targets multiple cancers and is reported to sensitize cancer drugs in MDR cancers. Although the compound showed promising activity in pre-clinical studies, its clinical use is limited due to limited water solubility and faster metabolism. To overcome this, several analogs of curcumin have been synthesized and tested pre-clinically in search of better therapeutic activity^[13,14]^. A large number of reported studies and increasing interest of researchers have reinforced the claim of curcumin being one of the most sought after natural product in the fight against cancer.

**Figure 1:**
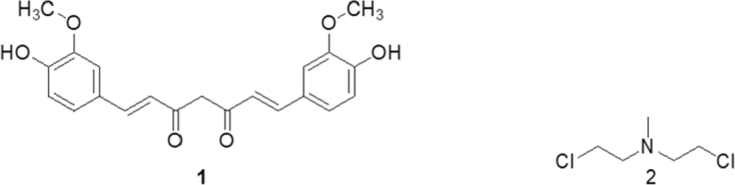
Structure of Curcumin and Nitrogen Mustard

Curcumin, structure [Strucuture 1, Figure 1], is a polyphenolic compound of up to 5% present in turmeric^[15]^. Turmeric is a traditional medicine and also used as a food additive. In the South Asian region, it is recognized as an important remedy in Ayurveda and Unani system of medicine and it belongs to the Zingiberaceae family^[16]^. Curcumin as a phytochemical, has been widely explored for its therapeutic potential through *in vitro* and *in vivo* investigations. It has been shown to possess biological activity against a large spectrum of physiological conditions, which include antioxidant^[17-20]^, chemo-protective^[21-23]^, anti-diabetic^[24-26]^ and anti-proliferative activity against cancer cells^[27-31]^.

## Developments in chemotherapy

**Figure 2:**
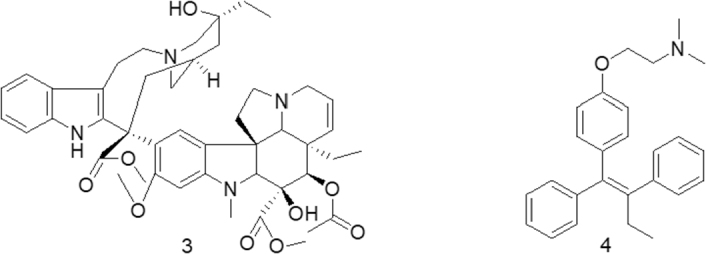
Structure of Vinblastine and Tamoxifen

Progress and milestones achieved in cancer chemotherapy in almost eight decades are historical in terms of their impact. Starting from a very non-specific cytotoxic agent, nitrogen mustard [Strucuture 2, Figure 1] in the early 1940s^[32]^ to revolutionizing studies on vinca alkaloids like vinblastine [Strucuture 3, Figure 2] in 1968^[33]^, which shifted the focus towards the use of natural products against various types of cancers. Although accidental, a remarkable discovery of estrogen receptor-specific activity of tamoxifen [Strucuture 4, Figure 2] in 1980 was another accomplishment of chemotherapy^[34]^ which led to more investigations directed towards target specific designing of anti-cancer agents. More insights into system biology led to the development of monoclonal antibody (MAB) for cancer treatment and consequential FDA approval to rituximab^[35]^ against B-Cell Lymphoma in 1997. The same year witnessed the arrival of 2-phenylaminopyrimidine derivative showing inhibitory effects on Abl-protein tyrosine kinase, which is responsible for the promotion of cell growth signaling pathway^[36]^.

**Figure 3:**
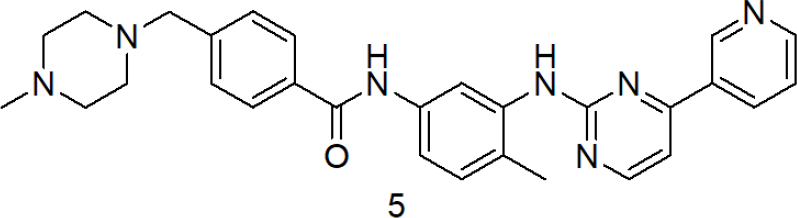
Structure of Imatinib

In 2001, the said derivative, which is now known as imatinib [Strucuture 5, Figure 3], obtained the recognition of the first FDA approved tyrosine kinase inhibitor, for the treatment of chronic myeloid leukemia. However, despite these commendable advancements in chemotherapy, cancer treatment remains a serious challenge due to drug-resistant cancers.

## Cancer drug resistance

The cancers not responding to chemotherapeutic treatment are termed as drug-resistant cancers. Cancer cells may get directly or indirectly protected against chemotherapy through different factors and means like epigenetics^[37-41]^, increased drug efflux by ATP-driven drug transporters^[42-44]^, DNA-damage repair^[45,46]^, anti-apoptotic mechanism^[47-49]^ and epithelial-mesenchymal transition (EMT)^[50-52]^.

### Curcumin against resistant breast cancer

There have been systematic efforts to identify drug-resistant cancers and formulate therapeutic solutions. The investigations involving Curcumin in this pursuit have resulted in promising results so far. Adriamycin resistant MCF-7_ADR_ and Tumor Necrosis Factor resistant BT-20_TNF_ breast cancer cell lines showed 15% (± 6%) and 8% cell viability respectively against curcumin at a dose of 1 μg/ml (2.7 μM)^[53]^. The same study claimed that curcumin exhibited the growth inhibitory effect on estrogen-dependent MCF-7 and T-47D as well as estrogen-independent SK-BR3 cell lines at lower concentrations, and arrested the majority of cells in the G2/M phase and inhibition of ornithine decarboxylase (ODC) activity. A comparative study of the effect of curcumin on human mammary epithelial (MCF-10A) and MDR breast carcinoma (MCF-7/TH) cell lines reported that the IC_50_ value of curcumin against MCF-10A was 3.5 times higher than that of MCF-7/TH although cytometric analysis showed equal accumulation of curcumin in both cell lines and it is well complemented with the apoptosis studies where 40 µM (24 hr) concentration of curcumin led 1.8% of MCF-10A cells into apoptosis while 46.6% of MDR, MCF-7/TH went into apoptosis under similar conditions, which in terms of considering the collateral damages is a significant observation^[54]^. In an investigation undertaken by Meiyanto *et al.*^[55]^, doxorubicin-resistant breast cancer cell lines MCF-7/Dox cells with over-expression of HER2 were tested against doxorubicin (IC_50_ = 7) and curcumin (IC_50_ = 80 ± 2.39) separately and in combination. The MTT Assay showed that curcumin at half of its IC_50_ concentration in combination with doxorubicin at half of its IC_50_ concentration, decreased the percentage cell viability of MCF-7/Dox cell lines by almost 80%, and this synergistic action of combinatorial treatment-induced cell death, evident through the accumulation of more cells in sub-G1 and G1 phase as compared to the percentage of cells when they were treated separately by doxorubicin and curcumin. Efficacy of curcumin against resistant breast cancer cell lines was demonstrated through SRB assay on MCF-7, antiestrogen-resistant MCF-7/LCC2 and MCF-7/LCC9 cell lines, which revealed IC_50_ values of curcumin to be 9.7, 12.2 µM and 11.34 µM respectively against these cancer cell lines and colony formation for each cell line was suppressed by curcumin at a concentration of 30 µM. These activities of curcumin were attributed to lowering of anti-apoptotic expressions and inhibition of NF-κB and Akt/mTOR pathway^[56]^. The photosensitization of cancer cells by curcumin towards photodynamic therapy (PDT) has been covered by Muniyandi *et al.*^[57]^ and apoptosis is the mode of action in majority of the works cited in the review. The adriamycin resistant breast cancer cell line MCF-7/ADR was found to be equally affected as MCF-7 cells (Cell viability 50%) on 45 minutes preincubation with curcumin (7.5 μM) followed by irradiation with blue light (450 nm, 100 mW/cm^2^) for 5 min and subsequent 24 h incubation^[58]^.

### Curcumin against resistant colorectal cancer

*In vitro* studies on human colorectal cancer cell line HCT116 and its isogenic 5-fluorouracil (5-FU) resistant cell line HCT116R in a 3D model showed that curcumin potentiated the anti-proliferative activity of 5-FU against these cell lines through apoptosis and inhibition of formation of colonies, with suppression of NF-κB pathway^[59]^. HCT116R cells were chemo-sensitized to the action of 5-FU and IC_50_ of 5-FU was recorded as 0.1 μM when it was used in combination with 5µM concentration of curcumin. This synergistic combination increased the percentage of apoptotic cells by 56% in HCT116R cell lines. The molecular role of curcumin in apoptosis has already been shown in another report where it intensified the downregulation of anti-apoptotic BclxL and cell division favoring cyclin D1 protein caused by 5-FU in HCT116 and HCT116+ch3 (Complemented with chromosome 3) cell lines and inhibiting activation of IkBα kinase and its phosphorylation^[60]^. Chemo-sensitization of drug-resistant cancer cell lines by curcumin, towards a particular chemotherapeutic agent, has been reported in one more investigation involving oxaliplatin sensitive human colorectal adenocarcinoma HT29 Cells and its oxaliplatin resistant derived sub-line HTOXAR3 cells, which showed that combination of curcumin and oxaliplatin almost reversed the oxaliplatin resistance in HTOXAR3 cell line with oxaliplatin IC_50_ reaching to 10.6 ± 2.2 μM which is fairly comparable to HT29 cell line whereas IC_50_ has been noted as 8.45 ± 1.6 μM^[61]^. Mechanistic aspects in this investigation showed that curcumin effected reversal of oxaliplatin resistance at 20 µM concentration against the HTOXAR3 cell line through inhibition of oxaliplatin-induced activation of NF-κB pathway, which otherwise would have led to an increase in anti-apoptotic expressions like survivin and Bcl-2.

### Curcumin against resistant leukemia

Daunorubicin resistant CD34^+^ acute myeloblastic leukemia cell lines KG1a and Kasumi-1 showed inhibition in growth and clonogenicity to curcumin treatment in dose and time-dependent manner^[62]^. In this study, Curcumin showed an IC_50_ value of 35.7 µM against KG1a 23.5 µM against Kasumi-1 on 96 hr exposure and completely stopped colony formation at 20 µM concentration. The mechanistic investigations reflected the role of curcumin in activation of Caspase-3, down-regulation of Bcl-2 mRNA expression and reduction in mitochondrial membrane potential in addition to remarkable morphological changes like cell shrinking and nuclear condensation, which are characteristics of apoptosis. Another drug-resistant leukemia cell line HL60 responded to curcumin with 50% growth inhibition at 30 µM concentration^[63]^. Cell cycle studies in this experiment established apoptosis as the mechanism of action of curcumin and arrest of the cell cycle in the S-phase was also reported in the same study.

### Curcumin against resistant lung cancer

Human non-small cell lung cancer cell line A549, which showed 50% cell viability at a high dose of 10,000 U of interferon (IFN)-alpha (IFNα), was investigated to understand the resistivity of these cells against such a higher concentration of IFNα^[64]^. On treatment with one-tenth of the IC_50_ value, the A549 cells showed an increase in p50 (NF-κB1) and p65 (RelA) subunits of NF-κB with respect to time, in addition to an increase in Cox-2 expression. On pretreatment with curcumin, a dose-dependent decrease in these subunits was noticed in Western Blot Analysis and a decrease in Cox-2 expression was also noted. Thus, curcumin showed a remarkable decrease in NF-κB and Cox-2 activity in a dose-dependent manner with a maximum dose of 50 μM in IFNα resistant A549 cell lines and it increased the vulnerability of cells towards the cytotoxic activity of IFNα. The lower water solubility of curcumin has led to the use of methods like micelles and nanocapsules for effective permeation and release. Adriamycin resistant A549/ADR lung cancer cell line showed a reversal of resistance to doxorubicin on treatment with polymeric micelles loaded with doxorubicin and curcumin^[65]^.

### Curcumin against resistant prostate cancer

In many of the references cited in this review, the induction of apoptosis has been one of the modes of action of curcumin. PI3/Akt pathway, which promotes cell growth, proliferation, and survival, is inhibited by curcumin^[66,67]^. Mechanistic studies, carried out at subtoxic concentrations of curcumin in LNCap cells showed that pretreatment with curcumin sensitized the cells towards tumor necrosis factor-related apoptosis-inducing ligand (TRAIL) through inhibition of the NF-κB pathway of cell survival^[68]^. Castration-resistant prostate cancer cell (CRPC) line C4-2B, showed a promising response to chemo-sensitization towards remarkably low concentration dose of 10nM docetaxel on pretreatment with a combination of 5 μM curcumin and 5 μM nelfinavir^[69]^, commendably without much adverse effect on primary prostate epithelial cells. The molecular study revealed an increase in pro-apoptotic markers caused by endoplasmic reticulum (ER) stress and decrease in expressions associated with PI3K/AKT survival pathway like phosphorylated-AKT. This *in vitro* finding was complemented with *in vivo* studies in the xenograft model. Docetaxel resistant CRPC lines PC3 and DU145 when treated with curcumin and curcumin nanoparticles prepared by emulsification showed cytotoxic effects of curcumin^[70]^. The IC_50_ values of curcumin in nanoparticle form against Docetaxel-resistant CRPC PC3 and DU145 cells were 5.0 ± 0.7 μM and 12.1 ± 1.1 μM respectively, while free curcumin showed IC_50_ values of 20.9 ± 0.3 μM and 27.1 ± 1.4 μM against these resistant cell lines respectively, underlining the fact that better delivery methods can potentiate the cytotoxic action of curcumin. A summary of *in vitro* activities of curcumin against various cancer cell lines has been compiled in Table 1.

**Table 1:** *In vitro* activity of Curcumin (Cur) alone and in combination

Cancer type	Cell line	Concentration in µM of Cur and other agents if any	*Cell viability as % of control	Ref. No.
Breast cancer	MCF-7	2.7 Cur	9 ± 1	[53]
	MCF-7_ADR_ (Adriamycin-Resistant)	2.7 Cur	15 ± 6	
	BT-20	2.7 Cur	1 ± 0	
	BT-20_TNF_ (Tumor Necrosis Factor-Resistant)	2.7 Cur	8 ± 0	
	MCF-10A (Normal Mammary Epithelial)	55.0 ± 3.53 Cur	50	[54]
	MCF-7/TH (Multidrug-Resistant)	17.5 ± 1.76 Cur	50	
	MCF-7	109 ± 1.915 Cur	50	[55]
	MCF-7/Dox (Doxorubicin-Resistant)	80 ± 2.39 Cur	50	
	MCF-7/Dox (Doxorubicin-Resistant)	7 Doxorubicin	50	
	MCF-7/Dox (Doxorubicin-Resistant)	40 Cur + 3.5 Doxorubicin	80	
	MCF-7	9.7 Cur	50	[56]
	MCF-7/LCC2 (Antiestrogen-Resistant)	12.2 Cur	50	
	MCF-7/LCC9 (Antiestrogen-Resistant)	11.34 Cur	50	
	MCF-7	7.5 Cur + PDT	50	[58]
	MCF-7/ADM (Adriamycin-Resistant)	7.5 Cur + PDT	50	
Colorectal Cancer	HCT116	9 Cur	50	[59]
	HCT116R (5 FU-Resistant)	5 Cur	50	
	HCT116	5 5-FU	50	
	HCT116R (5 FU-Resistant)	10 5-FU	>80	
	HCT116	5 Cur + 0.1 5-FU	50	
	HCT116R (5 FU-Resistant)	5 Cur + 2 5-FU	50	
	HCT116	20 Cur	50	[60]
	HCT116+ch3 (Complemented with chromosome 3)	5 Cur	50	
	HCT116	5 5-FU	50	
	HCT116+ch3 (Complemented with chromosome 3)	1 5-FU	50	
	HCT116	5 Cur + 1 5-FU	50	
	HCT116+ch3 (Complemented with chromosome 3)	5 Cur + 0.1 5-FU	50	
	HT29	8.5±1.6 Oxaliplatin	50	[61]
	HT29	9 ± 1.4 Cur	50	
	HT29	4.6 ± 1.1 Cur + 3.24 ± 0.7 Oxaliplatin	50	
	HTOXAR3 (Oxaliplatin Resistant Derived Sub-line of HT29)	8.3 ± 0.8 Cur	50	
	HTOXAR3 (Oxaliplatin Resistant Derived Sub-line of HT29)	30.2 ± 4.2 Oxaliplatin	50	
	HTOXAR3 (Oxaliplatin Resistant Derived Sub-line of HT29)	10 Cur + 10.6 ± 2.2 Oxaliplatin	50	
Leukemia	KG1a	35.7 Cur	50	[62]
	Kasumi-1	23.5 Cur	50	
	HL60	30 Cur	50	[63]
Lung Cancer	A549	1.89 Doxorubicin	50	[65]
	A549	2.6 Cur + 1.1 Adriamycin	50	
	A549/ADR (Adriamycin-Resistant)	69.7 Doxorubicin	50	
	A549/ADR (Adriamycin-Resistant)	98.5 Cur + 41.7 Adriamycin	50	
Prostate Cancer	C4-2B	0.59 Docetaxel	50	[69]
	C4-2B	30 Nelfinavir	50	
	C4-2B	59 Cur	50	
	C4-2B	0.01 Docetaxel+5 Nelfinavir+5 Cur	≈30	
	PC3	21.4 ± 0.8 Cur	50	[70]
	PC3 Docetaxel-Resistant	20.9 ± 0.3 Cur	50	
	DU145	19.5 ± 1.1 Cur	50	
	DU145 Docetaxel-Resistant	27.1 ± 1.4 Cur	50	

*% Cell viability as compared to control (control is 0% inhibition)

## Curcumin against cancer stem cells pathways

Cancer stem cells (CSCs) are those cells from the heterogeneous population of a tumor, which are capable of self-renewal^[69]^, aggressive proliferation, differentiation and cologenicity^[71-73]^. The aggressive characteristic properties of CSCs make them a bigger challenge than other neoplastic cells. Severe combined immunodeficiency disease (SCID) mice show new tumor growth when transplanted with CSCs^[74]^. An understanding of CSCs with respect to their properties, which are common to other cells and differentiating characters, which make them distinguished targets can lead to sophisticated strategies to deal with the CSCs. It has been shown that CSCs also show some regularity pathways similar to normal cells like Wnt/β-catenin^[75,76]^, Sonic Hedgehog (Hh)^[77,78]^, Notch^[76]^, and Hippo^[79,80]^ Signaling pathways.

### Curcumin against Wnt/β-catenin pathway

The role of the Wnt signaling pathway in cell multiplication and differentiation^[81,82]^ is well established. The irregular activation of Wnt pathway has been implicated in human cancer because of anomalous increase in the nuclear concentration of β-catenin and consequential activation of cancer associated genes^[83-85]^. β-catenin has been linked to various cancers including colorectal carcinoma, non-small cell lung cancer, breast cancer and prostate cancer^[86]^. Regulation of hyper-activated Wnt signally is an important strategy to control cancer involving this pathway. Human non-small cell lung cancer (NSCLC) cell line A427 and A549 on treatment with curcumin up-regulated micro RNA miR-192-5p and inhibited the cell growth and migration with the deactivation of Wnt/β-catenin pathway^[87]^. Similar deactivation by curcumin has been reported in medulloblastoma through regulation of the Wnt/β-catenin pathway with down-regulation of β-catenin and Cyclin D1^[88]^. Curcumin induced apoptosis in HCT-116 cells and derived cell lines without p53 or p21 genes at 20 μM with 67% to 88% cells arrested in G2/M phase and curcumin pretreatment inhibited transcriptional activity of the β-catenin/Tcf-Lef complex and activated Caspase-3^[89]^. Mechansitic insights from other investigations have consolidated the understanding of role of curcumin in inhibition of Wnt/β-catenin pathways in various cancers^[90,91]^.

### Curcumin against hedgehog signaling pathway

Sonic Hedgehog (SHH) signaling pathway involves transduction of signals directed at regulation of growth of multicellular organisms^[92,93]^. The growth and development in an embryo is a complex phenomenon and impaired SHH signaling is known to reflect in birth defects and complications pertaining to cancer growth^[94]^ in mammals.

Curcumin has been shown to regulate GLI1 mRNA and GLI1 reporter activity in prostate cancer cell lines and *in vivo* model and GLI1 is known to be an important transcription factor in Hedgehog pathway^[95]^. Significant outcomes were noticed in transforming growth factor-β1(TGF-β1)-stimulated PANC-1 cell lines, on treatment with curcumin (30 μM/mL), which not only inhibited the cell proliferation, invasion and migration but also reversed the EMT caused by TGF-β1. The down-regulated expressions of Shh and GLI1 in PANC-1 cells can be attributed to inhibition of Shh Signaling pathway by curcumin^[94]^. Many other studies on molecular mechanism of action have reported the reversal of EMT by curcumin through inhibition of Shh pathway^[96-98]^. Bladder cancer stem cells (BCSCs) UM-UC-3 and EJ, showed deactivation of the sonic hedgehog (Shh) signaling on treatment with 50 μM concentration of curcumin and increase in pro-apoptotic expressions of Bax and cleaved Caspase-3^[99]^. At the same concentration of curcumin, these cells considerably lost the capacity to form spheres and a higher percentage of cells was found in the G_0_/G_1_ phase. Shh protein and other downstream expressions in the hedgehog pathway like GLI1 and PTCH1, were found to be decreased in human primary medulloblastoma cells on treatment with curcumin^[100]^.

### Curcumin against notch signaling pathway

Notch signaling pathway is directly involved with cell proliferation and differentiation. Aberrant changes in this pathway lead to cancer and related features including drug resistance and tumor growth^[101,102]^. Gamma (γ)-secretase (GS) is an important component of Notch signaling and its inhibition is implicated in suppression of cancer growth, inhibition of tumor formation and subjugation of cancer stem cells^[103,104]^. Curcumin has been shown to restrain Notch pathway in esophageal cancer by suppressing GS components Nicastrin and Presenilin-1^[105]^. Notch signaling is known for enabling self-renewal and survival of cancer stem cells^[106]^. Curcumin has been shown to down-regulate the activated Notch-1 signaling human umbilical vein endothelial cells (HUVECs) exposed to hydrogen peroxide-induced cellular oxidative stress^[107]^. *In vivo* investigations on Sprague Dawley rats consuming high-fat diet showed that curcumin not only lowered the visceral fat, cholesterol and low-density lipoprotein but also suppressed the Notch-1 protein in liver cells^[108]^. The photosensitizing potential of curcumin in induction of apoptosis, inhibition of cell proliferation and regulation of Notch signaling has been demonstrated in cervical cancer cell line Me180. The cells incubated for 6 h with 1 μM solution of DAPT {N -[N- (3, 5-difluorobenzene acetyl-l-propionyl)]-(S)-phenylglycine tert-butyl}, which is a Notch receptor blocker and γ-secretase inhibitor in combination with 2.5 μM solution of curcumin were irradiated with laser (445 nm, 100 J/cm^2^). After 24 h, the group receiving curcumin and DAPT along with photodynamic treatment (PDT) showed increased apoptotic cell death and a remarkable inhibition rate of 79.27% in Notch1 mRNA expression whereas the group receiving DAPT alone and curcumin-PDT showed 39.99% and 32.33% inhibition rate respectively^[109]^.

## Curcumin: inroads into *in vivo*

The *in vitro* performance of any given compound is important for preliminary progress in drug development, however *in vivo* activity strengthens its claim of bearing the desirable therapeutic potential. Curcumin has been widely explored for its pharmaceutical potential for *in vitro* and *in vivo* investigations with remarkable outcomes. The concerns regarding poor water solubility (0.6 μg/mL) have been addressed through various methods to increase the bioavailability for *in vivo* studies. Successful efforts have been made through preparation of various formulations like nanoparticles^[110-113]^, liposomes^[114,115]^ and combinatorial treatment^[116-119]^ of curcumin with anticancer agents. It is important to note that free curcumin has also shown commendable activity during *in vivo* studies despite its limitations of lower water solubility, stability and bioavailability but the formulated curcumin or curcumin in combinatorial treatment has exceeded the performance of isolated treatment of free curcumin in majority of the cases.

Thus, a formulation overcoming challenges of solution stability, bioavailability, targeted delivery and lowest possible toxicity is much sought after in the case of every drug and curcumin is also not an exception in this regard. Many efforts have been successful in this direction and they have led to appreciable *in vivo* performance. A nano-suspension prepared from d-α-Tocopherol polyethylene glycol 1000 succinate and curcumin increased the water solubility by more than 400 times^[120]^ and the lyophilized nano-suspension, produced fine powder which could be again reconstituted by the addition of water.

The *in vivo* findings are not only important to ascertain the desirable pharmaceutical effect in a more natural environment but also to understand the pharmacodynamics, pharmacokinetics, toxic effects on vital organs like heart; kidney; liver and spleen while providing insights on mechanistic aspects of mode of action of a drug on histological and immunohistochemical examination of tissues. Careful recording of physiological and behavioral changes in the *in vivo* studies, changes in total body weight and survival time are important aspects of pre-clinical studies. The pre-clinical *in vivo* studies are crucial in the case of any molecule to prove its eligibility as a suitable candidate for advanced investigations.

### Curcumin against brain cancer *in vivo*

Subcutaneous and intracerebral orthotopic model of Human glioma U-87 cells in athymic mice on intraperitoneal dose of curcumin (120 mg/kg/day) showed less than 50% decrease in median tumor volume in subcutaneous xenograft while in the orthotopic model, the average life span of group receiving similar dose increased by 12% as compared to the control group^[121]^. In Female SCID mice xenograft model, human primary medulloblastoma cell*s* (DAOY) were subcutaneously injected and after 30 days, the animals were given oral gavage of curcumin (1 mg/kg) dissolved in corn oil^[122]^. The tumor growth inhibition in curcumin treated group was significantly noticeable as compared to the control group. The group of Smo/Smo transgenic medulloblastoma mice receiving oral dose of curcumin was reported to have a median survival time of 192 days as compared to the control group, which had a median survival time of 144 days. This observation is in agreement with earlier claims of ability of curcumin to cross Blood Brain Barrier^[123,124]^. Mechanistic insights in xenografted human medulloblastom D425 cells in athymic mice showed overexpression of p65 subunit of NF-κB and the curcumin treated group showed tumor growth inhibition which can be partially attributed to down regulation of p65 subunit^[125]^.

In another *in vivo* investigation, human glioblastoma U87-MG cells-inoculated nude mice were administered with 100 mg/kg per day of curcumin in DMSO in Phosphate Buffer Saline through intra-tumoral injections. After seven days, significant decrease in tumor size was observed in curcumin treated group. Microscopic examination post Acridine Orange staining showed increased acidic vesicular organelles in curcumin treated cells with intact nuclei, pointing towards curcumin-induced autophagy being responsible for cell deaths^[126]^.

### Curcumin against breast cancer *in vivo*

In the xenograft model of triple-negative human breast cancer MDA-MB-231 lines, curcumin showed reduction in tumor weight in a dose dependent manner and the most effective dose was reported as 200 μg/kg of curcumin administered through intraperitoneal injections for 28 days^[127]^. Transmission electron microscope images showed very distinct morphological changes related to apoptotic cell death without significant side effects on the animals. Oral dose of curcumin in combination with naringenin (20 mg/kg each) received by Swiss albino mice, transplanted with murine mammary Ehrlich ascites carcinoma (EAC) cells, resulted in reduction in the formation of new blood vessels in peritoneal and inner skin linings with reduction (80%) of total number of cells/mL in ascites fluid^[128]^. Athymic mice xenograft model of human triple-negative breast cancer MDA-MB-231 cells showed significant decrease in angiogenesis, cell proliferation and tumor size on treatment with 300 mg/kg/day intraperitoneal (i.p.) dose of curcumin^[129]^. Another xenograft model of triple negative breast cancer MBCDF-T cells in nude mice on combinatorial treatment of calcitriol (0.25 μg in 100 μL i.p., once a week) and curcumin (40 mg/kg daily in drinking water) showed inhibition of vascularization and tumor growth^[130]^.

Phosphorylated calixarene (POCA4C6)-encapsulated curcumin on intra-tumoral injection [providing (29.2 mg/kg)] showed strong inhibition of tumor growth in xenografted triple negative Breast cancer cell line BT-549 with indication of substantial early apoptosis as compared to the groups treated with empty micelles and free curcumin. Increased bioavailability through micelle-encapsulated delivery of curcumin was established with the help of increased fluorescence showing increased curcumin-accumulation in tumor^[131]^. MCF-7 xenograft subcutaneously inoculated in BALB/c nude mice when treated with a combination of paclitaxel and curcumin encapsulated in biodegradable polymeric nanoparticles prepared from tri-block copolymer of poly (ε-caprolactone)-poly(ethylene glycol)-poly(ε-caprolactone) showed highest tumor growth inhibition with insignificant loss in total body weight and highest survival percentage as compared to that of control group and groups treated individually with paclitaxel and curcumin^[132]^.

Human Breast cancer BT-474 cells with overexpression of HER-2, xenografted in athymic nude mice showed promising results with decreased mean tumor size as compared to other groups on treatment with 45 mg/kg dose of curcumin twice/week in combination with herceptin (2 mg/kg) once a week on intraperitoneal injection for four weeks^[133]^. A unique conjugate of curcumin was reported with membrane associated protein, Annexin A2 for the treatment of xenograft model of highly invasive Breast cancer cell line MCF10CA1a, which also showed overexpression of Annexin A2. PLGA nanoparticles loaded with Annexin A2-Curcumin conjugate at a dose of 20 mg/kg every alternate day for 32 days, decreased the tumor volumes by 44% ± 5.2% along with remarkable inhibition of neovascularization^[134]^. A subcutaneous xenograft model of triple negative breast cancer cell line 4T1 in Female Balb/c mice showed 27% reduction in the initial size of tumor on treatment with Curcumin loaded nanocompositen (NC) of Fe_3_O_4_ core and SiO_2_ shell on intra-tumoral injection of 40 μL of NC (20 μg curcumin, 0.46 mg/mL) followed by irradiation with a blue diode laser (450 nm, 150 mW/cm2) for 3 minutes and then second irradiation with near infrared laser (808 nm, 0.5 W/cm2) for 7 minutes on every alternate day for two weeks^[135]^. The observations are significant in this study considering availability of fewer treatment options in the case of triple negative breast cancer.

### Curcumin against Cervical Cancer *In Vivo*

Exosomes-extra cellular vesicles - as drug carriers are being considered for *in vivo* administrations. A formulation of curcumin with bovine milk-derived exosomes (Exocur) with a curcumin load of 18%-24% increased the bioavailability in various tissues. Human Cervical cancer (CaSki) xenograft in immunodeficient athymic female nude mice on treatment with 20 mg/Kg of Exocur decreased the tumor volume by 61% while free curcumin showed no effect on tumor size^[136]^. The same study reported no toxic effect of Exocur on mice on oral administration for two weeks at a dose of 2.5 mg/kg.

Liposomal curcumin (intraperitoneal dose of 25 mg/kg on alternated days) sensitized the human cervical cancer HeLa cell xenograft in non-obese diabetic SCID mice and 3-methylcholanthrene-induced cervical multistage squamous carcinoma in Swiss albino mice to Paclitaxel (intraperitoneal dose of 10 mg/kg, twice in a week) treatment. The combinatorial treatment of curcumin and paclitaxel reduced the tumor incidence by 76 percent as compared to control group whereas groups individually treated with curcumin and paclitaxel showed reduction of 40% and 56% respectively. Mechanistic observations showed that NF-κB activation caused by paclitaxel was suppressed on combinatorial treatment of curcumin and helped in inhibition of tumor growth in xenograft tumors^[137]^.

### Curcumin against colorectal cancer *in vivo*

The BALB/c nude mice xenograft model of human colorectal carcinoma cells HCT116, where HCT116 cells were separately transfected with pre-mRNA processing factor 4B (PRP4) and pre-mRNA processing factor 8 (PRP8), on treatment with intraperitoneally-injected 50 mg/kg of curcumin showed that the overexpressed PRP8 could not resist the curcumin-induced apoptosis as evident from tumor size and Western blot analysis showing overexpressed PRP8. PRP4 was hypothesized to resist the curcumin-induced apoptosis due to its kinase domain on the basis of earlier investigations and tumor growth did not get affected, which was also complemented with overexpressed PRP4 shown in western blot analysis. Further confirmations of hypothesis were made after deletion of kinase domain from PRP4, where curcumin successfully regulated tumor size in P4K^-/-^ HCT116 xenograft. These mechanistic insights implicated the role of kinase domain of PRP4 along with apoptosis inducing potential of curcumin^[138]^.

Curcumin bonded to human serum albumin (HSA) nanoparticles not only showed 300 times increased water solubility but also showed 14 times higher accumulation in tumor on intravenous administration after 1 hour as compared to that of free curcumin in the HCT1116 xenograft. 66% tumor growth inhibition by curcumin loaded HSA nanoparticles as compared to 18% tumor growth inhibition by free curcumin can per partially attributed to increased solubility and bioavailability of curcumin due to curcumin-HAS nanoparticle formulation^[139]^. A remarkable formulation of turmeric with phosphatidylcholine (1:4), which is called Mervia, showed five times increased bioavailability of curcumin as compared to free curcumin. Mervia was granted a US Patent (No. WO2013176555A1) in 2017 and recognized under improved complexes and compositions containing curcumin category. A combinatorial treatment of Mervia mixed with diet and biweekly intraperitoneal injection of oxaliplatin (7.5 mg/kg) in human colorectal cancer HCT116 cells xenograft for three weeks in female nude mice showed highest decrease of 51% in tumor volume^[140]^. Sub-toxic doses of curcumin (50 mg/kg) in combination with oxaliplatin (25 mg/kg), when administered every alternate day for 22 days in the human LoVo colorectal cells xenograft model in nude mice, caused remarkable suppression of tumor growth with signatures of apoptotic cell deaths. This investigation underlines the significance of combinatorial therapeutic regimens, where tumor growth can be inhibited without collateral damages^[141]^.

Curcumin loaded on polymeric Nanomicelle (OA-400), prepared from poly ethylene glycol 400 (PEG400) and oleoyl chloride, exhibited remarkable chemo-preventive activity against carcinogenic azoxymethane-induced colon cancer (subcutaneous dose of 15 mg/kg of azoxymethane) in male Wistar rats on pretreatment before administration of azoxymethane. Examinations after a period of 18 weeks showed that the number of tumors and size (33% of control) were considerably less in the group pre-treated with curcumin-loaded Nanomicelle as compared to the control group and group pre-treated with free curcumin. The control group showed highest number of tumors with significantly larger dimensions as compared to other two groups^[142]^. However, safety concerns related to toxicity of OA-400 have not been addressed in this work in details. Administration through oral gavage of combinatorial doses of the formulation of essential turmeric oil with curcumin and tocotrienol-rich-fraction of mixture of isomera of vitamin E (5 mg/kg and 2 mg/kg respectively) in 100 μL of sesame oil in the HCT-116 xenograft in severe combined immunodeficient (SCID) mice showed nearly 3-fold decrease in tumor size at the end of 40 days as compared to the control group with 7 to 8 fold increase in bioavailability^[143]^.

### Curcumin against gastric cancer *in vivo*

BGC-823 xenograft tumors on combinatorial treatment of curcumin with 5-FU and oxaliplatin showed remarkable inhibition of tumor growth through apoptotic pathway suggested through elevated expressions of caspase-3, 8 and 9 as compared to the group receiving combination of 5-FU with oxaliplatin and the group receiving curcumin alone^[144]^. Another xenograft model of gastric cancer cell line SGC-7901 showed involvement of apoptotic cell death as the underlying mechanism induced by curcumin^[145]^. One more BGC-823 xenograft, on treatment with curcumin encapsulated in Pluronic F-127 micelle (25 mg/kg), reduced the tumor volume by 75% as compared to the control group and almost 50% as compared to the group receiving 25 mg/kg of free curcumin^[146]^. The xenograft model of human gastric cancer cell line SGC7901, showed 90.02% ± 5.65% tumor inhibition rate (calculated from tumor volumes in test and control groups) on co-treatment with etoposide and curcumin loaded on nanostructured lipid nanoparticles. Etoposide and curcumin alone loaded on nanostructured lipid nanoparticles showed 69.13% ± 3.86% and 66.75% ± 4.1% tumor inhibition^[147]^.

### Curcumin against head and neck cancer *in vivo*

Balb/c nu/nu mice, subcutaneously xenografted with head and neck squamous cell carcinoma (HNSCC) SSC40 tongue cells showed decrease in tumor volume in curcumin-pre-treated group with a dose of 15 mg/day through oral gavage. The pre-treated group showed no development of tumors in 4-nitroquinolineoxide-induced carcinogenic model in oral cavity^[148]^. A study of HNSCC CAL27 cells xenograft in nude mice showed that liposomal curcumin (50 mg/kg) intravenously administered on alternate days for a period of 35 days resulted in 71.8% reduction in mean tumor weight in well-established xenograft tumors^[149]^. The study also reported normal appearance of kidney, liver, heart, spleen and lung on histological examination and reduced expression of NF-κB (p65) was noted in the immunohistochemical post-treatment profiling of tumor cells.

2-hydroxypropyl-γ-cyclodextrin (HPγCD) inclusion complex-loaded phospholipid liposomes exhibited a very effective delivery of curcumin, which resulted in enhanced antiproliferative activity through apoptotic cell death in human osteosarcoma KHOS xenograft model on intra-tumoral injection of 20 μL solution of the said formulation on alternated days for two weeks^[114]^. The 10,000 fold increase in curcumin-water solubility on preparation of inclusion compound with HPγCD was reported in the said investigation, which is a remarkable enhancement in terms of addressing concerns regarding lower bioavailability of curcumin. Lipid nanoparticles loaded with doxorubicin and curcumin were prepared from biodegradable 1,2-Distearoyl-sn-glycero-3-phosphoethanolamine-Poly(ethylene glycol) (DSPE- PEG) polar lipid. BALB/c nude mice xenograft of KHOS was intravenously administered for three weeks with a frequency of two administrations each week providing 5 mg/kg of doxorubicin and 10 mg/kg of curcumin showed tumor growth inhibition ratio (TGI) of 81.3%, which was calculated as [(Weight of control - Weight of treated) ÷ Weight of control] × 100. The group receiving only doxorubicin and only curcumin showed TGI of 10.3 and 11.9 respectively^[150]^. Xenograft model of oral squamous cell carcinoma cells HSC-3 in female SCID mice showed decrease of 21% in tumor volume at the end of study as compared to the initial tumor volume, on treatment with the peptide hydrogel loaded with the combination of curcumin and doxorubicin (1 μM and 0.164 μM respectively), while the tumor grew up to 368% of the initial volume when the combination was administered through solution. This combinatorial treatment of *in vivo* xenograft model of head and neck cancer showed prevalently apoptotic cell death on histological examination^[151]^. Xenograft model of EC9706, esophageal squamous cell carcinoma (ESCC) showed larger decrease in tumor size in the group treated with 50 μM curcumin in combination with 5-FU (5 mg/Kg) as compared to control group and other group including the groups treated with curcumin and 5-FU alone, indicating the supporting role of curcumin in increasing the 5-FU-mediated apoptosis^[152]^.

### Curcumin against liver cancer *in vivo*

Xenograft model of hepatocellular carcinoma H22 cells in nude male mice, showed better inhibition of tumor growth on daily dose of 100 mg/kg curcumin through intragastric administration for 2 weeks as compared to control group^[153]^. The subcutaneous tumor xenograft of human hepatocellular carcinoma SMMC-7721 cells in BALB/c nude female mice showed minimum tumor volume and highest tumor inhibitory rate (TIR) of 70% on combinatorial treatment of curcumin (56.65 mg/kg) + 5-FU (10 mg/kg) as compared to the control^[154]^. Human hepatocellular carcinoma HepG2 cells xenograft tumor in female BALB/c nude mice showed more than 50% reduction in tumor volume on daily intraperitoneal administration of curcumin (200 mg/kg) for one month with indications of autophagy^[155]^. Group receiving the combinatorial treatment of metformin (150 mg/kg) with curcumin (60 mg/kg) in subcutaneous HepG2 xenograft in nude mice showed 58.3% inhibition of tumor growth as compared to that of group treated with 150 mg/kg of metformin and the group receiving only 60 mg/kg of curcumin^[156]^. The study reported insignificant decrease in total body weight. Orally administered dose (60 mg/kg) of micellar-curcumin solution in combination with cisplatin (intraperitoneal injection 1 mg/kg) showed inhibition of tumor growth in the xenografted model of HC-AFW-1 pediatric hepatocellular carcinoma with increase in concentration of curcumin in different organs and blood^[157]^.

An interesting *in vivo* experiment showed the antiproliferative action of FDA approved histone deacetylase inhibitor (HDACi)-Trichostatin A (TSA) and combinatorial treatment of curcumin in resisting the Trichostatin-induced metastasis. Xenografted tumor models of human hepatocellular cancer Hep23 cells and human lung cancer H23 cells in nude mice showed decrease in tumor weight and suppression of metastasis on combinatorial treatment of TSA(10 mg/kg/d) and curcumin (40 mg/kg/d)^[158]^. The findings rationalize the combinatorial therapeutic regimens in order to suppress the complications arising from the chemotherapeutic agents.

### Curcumin against pancreatic cancer *in vivo*

A combinatorial treatment of phenethyl isothiocyanate (0.3 mg), which is a dietary compound found in certain vegetables with curcumin (110.5 mg) in NCr nude mice xenografted with human pancreatic cancer PC-3 cells, showed significant growth inhibition at these lower concentrations at the end of 28 days, starting from the day when xenograft was established properly^[159]^. Another investigation on PC-3 xenograft on treatment with combinatorially encapsulated docetaxel and curcumin in biocompatible lipid polymer nanoparticles prepared from lecithin and PEG-DSPE, providing a dose of 5mg/kg docetaxel and 10 mg/kg curcumin, showed TIR of 82.5%, which was the highest as compared to the groups receiving only encapsulated docetaxel (5 mg/kg) and the group receiving encapsulated curcumin (10 mg/kg) alone with TIR of 45.2% and 62.1% respectively^[160]^.

### Curcumin against lung cancer *in vivo*

*In vivo* antitumor activity determined through intra-peritoneal administration of curcumin in a dose of 45 mg/kg against xenografted 801D highly metastatic human lung cancer, showed greater size reduction in curcumin-treated animals with very clear and intact tumor boundary, which is indicative of significantly decreased tumor invasiveness and the observations were supported by decrease in MMP-2 and MMP-9 expressions^[161]^. In another *in vivo* study, xenograft of 801D showed a decrease of 41% in average weight of tumors in curcumin-treated group with clearer boundaries along with weaker staining of Cdc42 as compared to the control group, hinting towards inhibitory effect of curcumin on tumor growth and invasion through suppression of CDc42^[162]^.

Orthotopic model of human lung cancer cells (A459) in athymic nu/nu mice was studied for combinatorial treatment of curcumin and doxorubicin loaded on Janus nanoparticles prepared from water-in-oil-in water double-emulsion solvent-evaporation method^[163]^. Janus nanoparticles prepared from FDA approved, biodegradable polymer Poly(lactic-co-glycolic acid) (PLGA) and FDA approved lipid, Precirol® ATO5, loaded with doxorubicin and curcumin when administered through aerosol inhalation technique showed the tumor size to be of six fold lesser as compared to untreated group after four weeks. In another well-established A549 xenograft, synergistic action of docetaxel (10 mg/kg) and curcumin (15 mg/kg), showed successful inhibition of tumor growth and metastasis^[164]^.

Xenograft model of gefitinib resistant human lung cancer cell lines CL1-5, A549 and H1975 in SCID mice showed sensitization of cancer cells on combinatorial treatment of gefitinib (60 mg/kg) and curcumin (1 g/kg) with increased tumor growth inhibition as compared to control and other groups^[165]^. The same study reported lesser damage to intestinal mucosa in the group treated with the combinatorial doses, through reduction of p38-activation, known to be caused by gefitinib as reported in other investigations. Xenografted model of gefitinib-resistant Non-Small Cell Lung Cancer cell lines H157 and H1299 on treatment with combination of orally administered curcumin (1 g/kg) with gefitinib (100 mg/kg), exhibited commendable decrease in tumor size indicating chemo-sensitizing potential of curcumin against gefitinib-resistant cancer^[166]^. Another combinatorial treatment of Mervia effectively restricted lung metastasis of mammary gland tumor cell line ENU1564 in athymic nude mice^[167]^. Combinatorial treatment of 500mg/kg of curcumin suspended in 10% Tween-80 with 200 mg/kg of Phospo-sulindac (PS) in the subcutaneous adenocarcinomic human alveolar basal epithelial cell (A549) xenograft resulted in 51% synergistic inhibition of tumor growth, which was 50% more effective than the PS treatment alone^[168]^. Another xenograft model of A549 on treatment with 20 mg/kg subcutaneous dose of lipid-polymer hybrid nanoformulation-encapsulating curcumin for seven weeks, decreased the tumor volume by 52.1% and substantial decrease in Annexin A2 as compared to the control group, whereas the group treated with liposomal curcumin showed 32.2% decrease in tumor volume^[169]^.

### Curcumin against Prostate cancer *in vivo*

*In vivo* subcutaneous xenograft of luciferase-expressed human prostate cancer cell line PC-3-Luc, on treatment with 100 mg/kg of curcumin showed decrease in tumor volume and significant suppression of tumor metastasis. Detailed profiling indicated decreased cellular levels of matriptase, which appears to be responsible for prevention of metastasis^[170]^. PC-3 xenograft in BALB/c nu/nu male mice on daily intraperitoneal injection of curcumin (100 mg/kg) for 30 days showed significant decrease in tumor growth as compared to the control^[171]^.

**Figure 4:**
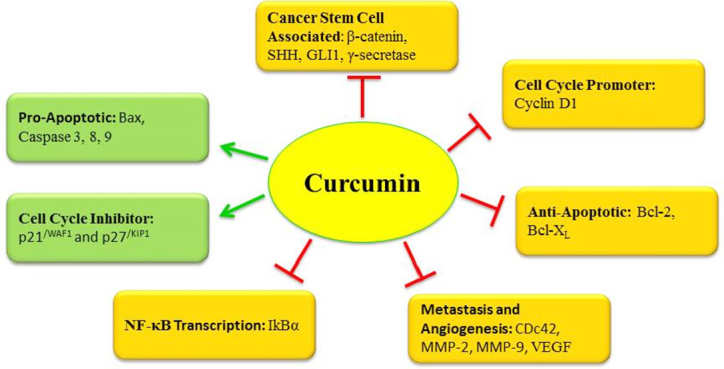
Summarized biological actions of curcumin

Castration-resistant prostate cancer (CRPC) C4-2B cells, xenografted in athymic nude mice showed promising result when a combinatorial dose of docetaxel (10 mg/kg), nelfinavir (20 mg/kg) and curcumin(100 mg/kg) was administered intraperitoneally, two weeks after subcutaneous injection of C4-2B cells. The group treated with combinatorial dose showed the suppression of the tumor growth by 3.5 folds as compared to the group treated with docetaxel alone and the vehicle-treated control group showed 22 folds increase in tumor after a period of 4 weeks^[69]^. In another report on human prostate cancer xenograft with C4-2B cells in nude mice, intra-tumoral injection of curcumin (25 µg/mouse) in DMSO inhibited the tumor growth by more than 50% with increased levels of β-catenin. Silencing of expression of protein kinase D1 (PKD1) by using relevant SiRNA pretreatment before the curcumin dose showed substantially decreased levels of PKD1 expressions as well as lower levels of β-catenin on the cell surface^[172]^, which points towards probable role of PKD1 in overexpression of β-catenin and curcumin mediated inhibition of tumor growth. A xenograft model of DU-145, an androgen-independent prostate cancer cell line in SCID mice was treated through oral gavage with a solution of curcumin (equivalent to 5 mg/kg), 0.5% methylcellulose and 0.1% Tween 80 and it showed 89% decrease in occurrence of metastatic tumors and remarkable decrease in average range of tumor volumes^[173]^. Balb/c nude mice xenograft model of androgen-dependent, TNF-related apoptosis-inducing ligand (TRAIL)-resistant human prostate cancer LNCaP cells was investigated to understand the underlying mechanism of growth inhibitory effects of curcumin. Oral injection of combinatorial treatment of curcumin 30 mg/kg and TRAIL (15 mg/kg), sensitized the TRAIL-resistant LNCaP, which was evident through tumor growth inhibition and suppression of metastasis. The immunohistochemical study on xenografted tumors, undertaken after six weeks of combinatorial treatment, showed a significant decrease in expression of MMP-2, MMP-9 and VEGF staining as compared to the control and pronounced expression of cell cycle inhibitors, p21^/WAF1^ and p27^/KIP1^. Westen blot showed intensified bands of pro-apoptotic Bax-expressions and down-regulated expressions of anti-apoptotic Bcl-2 and Bcl-X_L_^[174]^. A summary of *in vivo* activities of curcumin against various cancers has been compiled in Table 2.

**Table 2:** *In vivo* activity of Curcumin (Cur)

Cancer type	Cell line used in xenograft model	Dose in mg/kg body weight and formulation with other agent if any	Mode of administration	**Percentage inhibition in tumor growth as compared to control	Ref. No.
Brain cancer	U-87	120 Cur	Intraperitoneal	50	[121]
	DAOY	1 Cur	Oral gavage	≈ 62	[122]
	U87-MG	100 Cur	Intra-tumoral	≈ 50	[126]
Breast cancer	MDA-MB-231	0.2 Cur	Intraperitoneal	≈ 30	[127]
	MBCDF-T	40 Cur + 0.00025 calcitriol	Oral + intraperitoneal	≈ 80	[130]
	MCF10CA1a	20 Conjugate of Annexin A2-Cur	Intravenous	44 ± 5.2	[134]
	4T1	Dose in mg/kg body wt. not available 40 μL Cur loaded Fe_3_O_4_-SiO_2_ nano-composite (Cur 0.46 mg/mL)	Intratumoral	27	[135]
Cervical	CaSki	20 Cur loaded Exosome	Oral gavage	61	[136]
	HeLa	25 Cur + 10 Paclitaxel in lysosome	Intraperitoneal	70	[137]
Colorectal	HCT1116	20 Cur loaded on HSA	Intravenous	66	[139]
	LoVo	50 Cur + 25 Oxaliplatin	Intraperitoneal	≈ 43	[141]
	HCT1116	5 Cur + 2 tocotrienol rich mixture of Vit E	Oral gavage	≈ 70	[143]
Gastric	BGC-823	25 Cur in Pluronic F-127 micelle	Intraperitoneal	50	[146]
Head and neck	SSC40	15 Cur	Oral gavage	≈ 70	[148]
	CAL27	50 Cur in Liposome	Intravenous	71.8	[149]
	KHOS	10 Cur + 5 Doxorubicin loaded in Nanolipid	Intravenous	81.3	[150]
Liver	SMMC-7721	56.65 Cur + 10 5-FU	Intraperitoneal	70	[154]
	HepG2	200 Cur	Intraperitoneal	≈ 50	[155]
	HepG2	60 Cur + 150 Metformin	Intraperitoneal + oral	58.33	[156]
Pancreatic	PC-3	110.5 Cur + 0.3 phenylisothiocyanate	Intraperitoneal	≈ 76	[159]
	PC-3	10 Cur + 5 Docetaxel loaded on Nanolipid	Intravenous	82.5	[160]
Lung	801D	60 Cur	Intraperitoneal	≈ 50	[161]
	A459	15 Cur + 10 Docetaxel	Intravenous	≈ 70	[164]
	CL1-5	1000 Cur + 60 Gefitinib	Oral	≈ 70	[165]
	H1299	1000 Cur + 100 Gefitinib	Oral gavage	≈ 50	[166]
	H157	1000 Cur + 100 Gefitinib	Oral gavage	≈ 50	[166]
	A459	500 Cur + 200 Phospo-sulindac	Oral gavage	51	[168]
Prostate	PC3-Luc	100 Cur	Intraperitoneal	≈ 40	[170]
	C4-2B	100 Cur + 20 Nelfinavir + 10 Docetaxel	Intraperitoneal	≈ 90	[171]
	DU-145	5 Cur	Oral gavage	≈ 40	[174]
	LNCaP	30 Cur + 15 TRAIL	Oral	≈ 50	[175]

**Percentage inhibition of tumor growth as compared to control (control is 0% inhibition)

## Curcumin: the clinical trials

Curcumin has been recognized and granted FDA approval for clinical trials in determination of dose and as an adjuvant along with the major chemotherapetutic regimen. A clinical trial undertaken on healthy volunteers to understand the pharmacokinetics of curcumin in 12 volunteers (5 Males and 7 Females) between ages 18-65 years with a randomized dosage of 50% receiving single dose of 10g and the remaining 50% receiving 12 g of powdered extract of turmeric in capsules^[175]^. Reverse phase HPLC used for quantification showed no free curcumin in serum except for one candidate and high rate of covalent bonding of glucuronic acid and sulfation at phenolic -OH group in curcumin was observed. The adverse symptoms reported in this study were headache in one candidate, while one more candidate reported sore arms. However these symptoms are most unlikely to be caused by curcumin treatment. Three month long oral treatment of 25 high risk patients with different conditions including a gradual increase of 500 mg/day of curcumin upto 8 g/day, showed no adverse effects which could be associated with curcumin administration^[176]^. The serum level of curcumin was found to decrease with respect to time and the highest level of serum curcumin was detected within one or two hours of oral ingestion, where the patient receiving 8 g/day dose of curcumin showed 1.77 ± 1.87 μM curcumin in serum. The study showed that upto 8 g/day dose of curcumin did not show adverse effects and also showed improvement in precancerous lesions on histological examination.

A formulation of curcumin, Theracurmin tested on healthy volunteers showed improved bioavailability of curcumin with a peak plasma level of 0.74 ± 0.18 μM and t_1/2_ of 13.3 h for a single dose of 210 mg^[177]^. Theracurmin was administered in a group of 16 patients-undergoing gemcitabine-based chemotherapy for pancreatic cancer- in the form of 100 mL flavored drink with an initial dose of 200mg followed by doubling of dose in the absence of adverse side effects. The peak plasma levels was found to be 1.19 μM with a dose of 400 mg/day. The patients receiving Theracurmin showed improvement on adverse effects caused by chemotherapy and improvement in scores of quality-of-life^[178]^.

Healthy volunteers used formulation of curcumin in the form of gum administered over a period of 1h with chewing and keeping the gum for 4 minutes against buccal mucosa. The peak serum concentration was recorded to be 0.45 ± 0.17 μM at 4 h with decreased levels of pro-inflammatory biomarkers TNF-α with ~ 2 g of released curcumin^[179]^.

Eleven patients 7 males and 4 females in the age group of 12-26 years, suffering from advanced stage of metastatic osteogenic sarcoma and 6 healthy volunteers on treatment of solid-lipid particles loaded with curcumin in capsule form showed increased levels of curcumin in plasma and decreased level of TNFα^[180]^. No adverse effects were noticed in these patients, which could be correlated with curcumin consumption. The plasma levels of curcumin were constantly found to be in the range of 0.11 ± 0.024 μM in patients on treatment with the nanoformulation. In a Phase II clinical trial with 25 patients (median age 65 year) suffering from advanced pancreatic cancer already undergone surgery, radiotherapy or chemotherapy were treated with a daily oral dose of curcumin (8 g) in caplets for 8 weeks^[181]^. One patient showed a brief reduction of 73% in tumor and no adverse effect in all the patients and the peak plasma level of curcumin remained in the range 0.059 to 0.11 μM for the one month duration. A summary of clinical trials of curcumin has been compiled in Table 3.

**Table 3:** Clinical trials of Curcumin

Group size	Formulation	Health status of volunteers	Dose per day	Average peak serum/plasma concentration in μM	Remarks	Ref.
12	Powdered extract of curcuminoids in capsule	Healthy	10 g (N = 6) 12 g (N = 6)	No free curcumin was detected in plasma	No adverse side effects	[175]
25	Curcumin powder	Patients with one of the following conditions a) recently resected urinary bladder cancer b) arsenic Bowen’s disease of the skin c) uterine cervical intraepithelial neoplasm d) oral leukoplakia e) intestinal metaplasia	4 g 6 g 8 g	Peak serum level (PSL) 0.51 ± 0.11 0.63 ± 0.06 1.77 ± 1.87	No adverse effect and improvement in precancerous lesions	[176]
06	Theracurmin (nanoparticle formulation)	Healthy	150 mg and 210 mg	Peak plasma level (PPL) 0.74 ± 0.18	No adverse effect except for one report of diarrhea in one volunteer after a single oral dose of 150 mg	[177]
16	Theracurmin (nanoparticle formulation) in 100 mL flavored drink	Patients receiving gemcitabine based therapy for pancreatic cancer or biliary tract cancer	200 mg (N = 10) 400 mg (N = 06)	PPL 0.87 1.19	Improvement in adverse effect of chemotherapy	[178]
10	Curcumin in chewing gum	Healthy	2 g	PSL 0.45 ± 0.17	Decreased levels of pro-inflammatory marker TNF-α is observed	[179]
17	Solid-lipid particles loaded with curcumin in capsule	06 Healthy 11 Patients with metastatic osteogenic sarcoma	400 mg to 1.6 g	PPL at dose of 1.6 g 0.11 ± 0.024	No adverse effects	[180]
25	Caplet	Advanced pancreatic cancer already treated through surgery or radiotherapy or chemotherapy	8 g	PPL 0.059 to 0.11	No adverse effect in the participants and one patient showed brief reduction of 73% in tumor volume	[181]

## Conclusion

Curcumin has shown commendable potential during *in vitro* and *in vivo* studies against vrious cancers. It has also been established through clinical trials that curcumin does not show any adverse effect upto a daily dose of 8 g to 12 g. However, issues related to lower solubility, lower bioavailability and less stability in physiological conditions during human trials are yet to be resolved. Similar challenges have been overcome in case of taxol-a plant derived anticancer agent-which has now an FDA approved anticancer drug.

A biocompatible drug delivery system with a very safe toxicity profile may revolutionize the curcumin-based adjuvant chemotherapy with different roles in chemoprevention, chemo-sensitization and chemo-protection from adverse side effects of chemotherapeutic agents. The combinatorial treatment of curcumin has been shown to sensitize the drug-resistant cancer towards existing anticancer drugs and this aspect of combinatorial treatment can be exploited to fully utilize the curative potential of curcumin. In the near future, curcumin may play an important role in chemotherapeutic regimes against different types of cancers.

## Declarations

### Copyright

© The Author(s) 2021.
